# Sarcopenia and body fat change as risk factors for radiologic incisional hernia following robotic nephrectomy

**DOI:** 10.1007/s00256-023-04371-y

**Published:** 2023-05-30

**Authors:** Simin Hajian, Alireza Ghoreifi, Steven Yong Cen, Bino Varghese, Xiaomeng Lei, Darryl Hwang, Khoa Tran, Tapas Tejura, Gilbert Whang, Hooman Djaladat, Vinay Duddalwar

**Affiliations:** 1 Department of Radiology, University of Southern California, Los Angeles, CA USA; 2 Institute of Urology, Norris Comprehensive Cancer Center, University of Southern California, Los Angeles, CA USA

**Keywords:** Incisional hernia, Nephrectomy, Sarcopenia, Abdominal visceral fat, Abdominal subcutaneous fat

## Abstract

**Objective:**

To assess the effect of body muscle and fat metrics on the development of radiologic incisional hernia (IH) following robotic nephrectomy.

**Materials and Methods:**

We retrospectively reviewed the records of patients who underwent robotic nephrectomy for kidney tumors between 2011 and 2017. All pre- and postoperative CTs were re-reviewed by experienced radiologists for detection of radiologic IH and calculation of the following metrics using Synapse 3D software: cross-sectional psoas muscle mass at the level of L3 and L4 as well as subcutaneous and visceral fat areas. Sarcopenia was defined as psoas muscle index below the lowest quartile. Cox proportional hazard model was constructed to examine the association between muscle and fat metrics and the risk of developing radiologic IH.

**Results:**

A total of 236 patients with a median (IQR) age of 64 (54–70) years were included in this study. In a median (IQR) follow-up of 23 (14–38) months, 62 (26%) patients developed radiologic IH. On Cox proportional hazard model, we were unable to detect an association between sarcopenia and risk of IH development. In terms of subcutaneous fat change from pre-op, both lower and higher values were associated with IH development (HR (95% CI) 2.1 (1.2–3.4), *p* = 0.01 and 2.4 (1.4–4.1), *p* < 0.01 for < Q1 and ≥ Q3, respectively). Similar trend was found for visceral fat area changes from pre-op with a HR of 2.8 for < Q1 and 1.8 for ≥ Q3.

**Conclusion:**

Both excessive body fat gain and loss are associated with development of radiologic IH in patients undergoing robotic nephrectomy.

## Introduction

Incisional hernia (IH) is defined as a protrusion of abdominal fat/viscera or omentum through the abdominal wall at the site of a surgical incision [1]. It is a frequent complication following abdominal surgeries with an incidence ranging between 3 and 20% following open surgical procedures [2]. The increasing use of minimally invasive surgeries in recent years has been associated with an increased rate of IH up to 25% and the emergence of new types of IHs, such as trocar site hernia [3, 4]. IH may present with discomfort, pain, and bulge, as well as bowel incarceration and/or strangulation that can impose a serious risk of morbidity and mortality [5, 6]. Patients with IH experience decreased quality of life due to lower physical and social functioning, general health perception, cosmesis, and body image [2, 7]. Several studies have been performed to understand the risk factors of IH, yet limited data is available on the role of musculoskeletal metrics.

Body composition features such as fat and muscle metrics have been shown to be associated with treatment outcomes [8–10]. Several measurements (e.g., body mass index (BMI), waist circumference, and waist to hip ratio) have been used conventionally to assess these features [11]. However, prior studies have argued that these parameters are not appropriate enough for the evaluation of musculoskeletal characteristics [12]. On the one hand, sarcopenia, as a marker of chronic muscle depletion and frailty, has been increasingly explored as a predictor of adverse surgical outcomes (e.g., perioperative complications and long-term mortality), especially in cancer patients [8–10]. It can be easily measured by computed tomography (CT) scan using indices such as total/psoas muscle skeletal cross-sectional area at the level of L3 or L4 [13, 14]. On the other hand, high visceral fat or visceral obesity have also been shown to be associated with increased postoperative complications [15, 16]. Taken together, both body muscle and fat metrics may potentially affect hernia development due to weakness of the abdominal wall and/or increase in the abdominal wall tension. However, the association between sarcopenia/body fat change and IH is poorly understood. Very few studies, all in the field of general and colorectal surgery, are available on this topic with conflicting results [12–14, 17, 18].

In this study, we aimed to assess the effect of sarcopenia and body fat change on the incidence of radiologic IH following robotic nephrectomy. This will help in establishing a two-way bench- to bed-side communication that will result in a more appropriate decisions by the clinicians and patients and eventually improved clinical outcomes.

## Materials and Methods

### Study design and population

In this multidisciplinary cross-sectional study, we retrospectively reviewed the records of consecutive patients who underwent robot-assisted partial or radical nephrectomy for renal cancer at our institution between January 2011 and April 2017. The nephrectomies were performed using da Vinci® surgical system (Intuitive Surgical, Inc, CA). The exclusion criteria were: (1) patients without a preoperative abdominopelvic CT scan performed within 6 months prior to surgery, (2) history of abdominal surgery with mesh repair, (3) abdominal operation during the follow-up, (4) patients with missing postoperative imaging studies, and (5) patients with severe body deformity in whom appropriate calculation of musculoskeletal metrics were not possible. This study was approved by our institutional review board (no: HS-036031) and performed in accordance with the ethical standards of the 1964 Declaration of Helsinki and its subsequent amendments. Written informed consent was obtained from all patients in the study.

### Imaging protocol

All preoperative CT scans were performed within 6 months before surgery. Postoperative scans were done every 3–6 months in the first 2–3 years and then annually for routine oncological follow-up of renal cancer, according to the National Comprehensive Cancer Network (NCCN) guidelines [19]. CT scans were done either at the University of Southern California or outside facilities using multiple scanners and imaging protocols; however, only triple-phase abdominopelvic scans (i.e., without contrast, arterial, and venous) that covered the areas of interest were included in this study.

### Radiological assessment

**Fig. 1:**
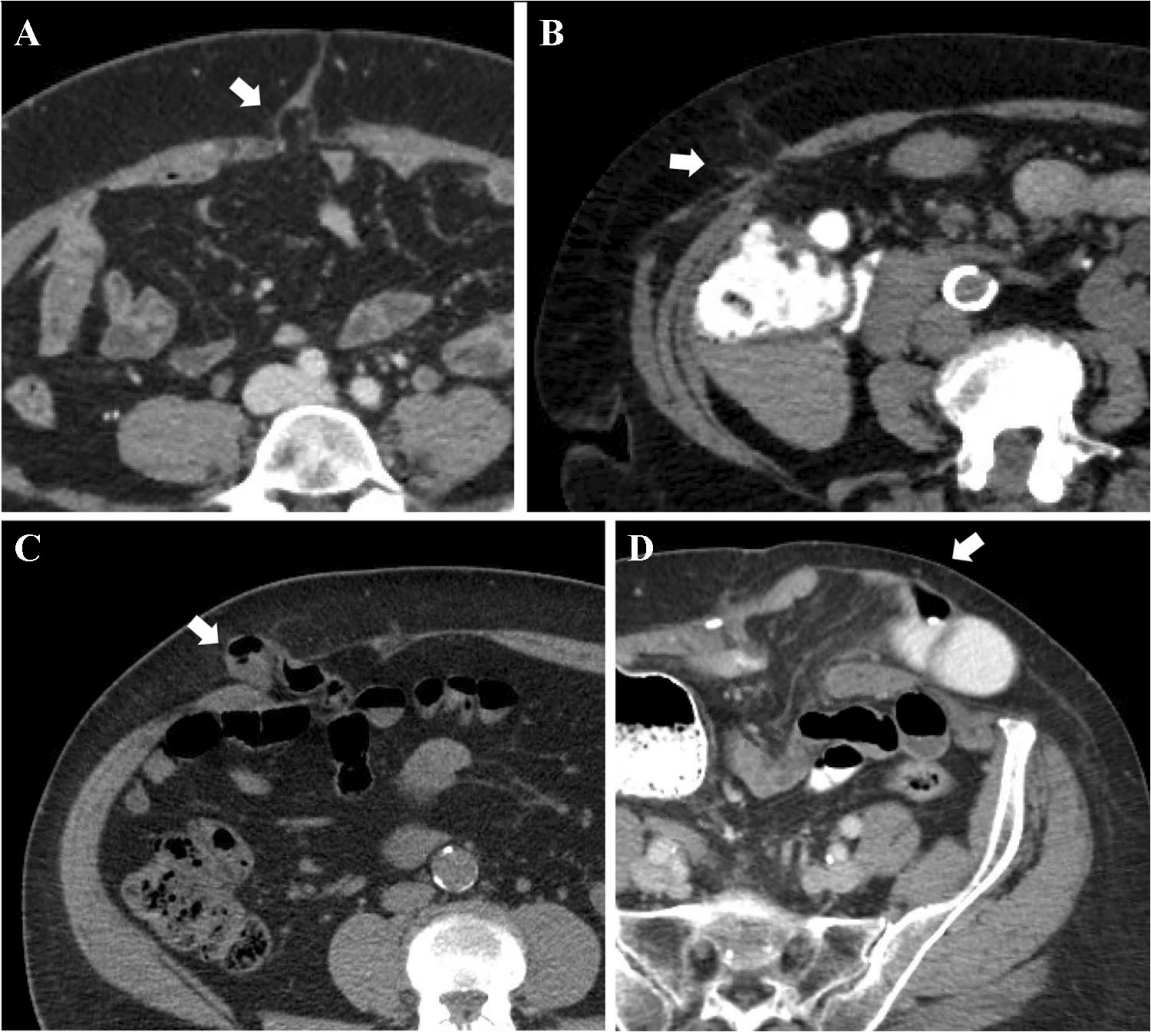
Different types of incisional hernia based on the Tonouchi classification: (**A**) early onset, (**B**) late onset, (**C** and **D**) bowel containing: white arrows show the hernia locations

All pre- and post-operative imaging were re-reviewed by expert genitourinary radiologists (GW, KT, and TT, supervised by VD), who were blinded to the clinical data, to detect IH. A two-way mixed with absolute agreement intraclass correlation coefficient (ICC3,1) of 0.94 (95% CI 0.86, 0.97) was observed among three radiologists who independently reviewed the CT images. Radiologic IH features, including size, location, and type, were recorded. Size of IH was calculated at the maximum fascial defect in axial CT view and type of IH was classified based on Tonouchi classification as early-onset, late-onset, and bowel/fat containing types (Fig. 1) [20].

### Quantitative image analysis

**Fig. 2:**
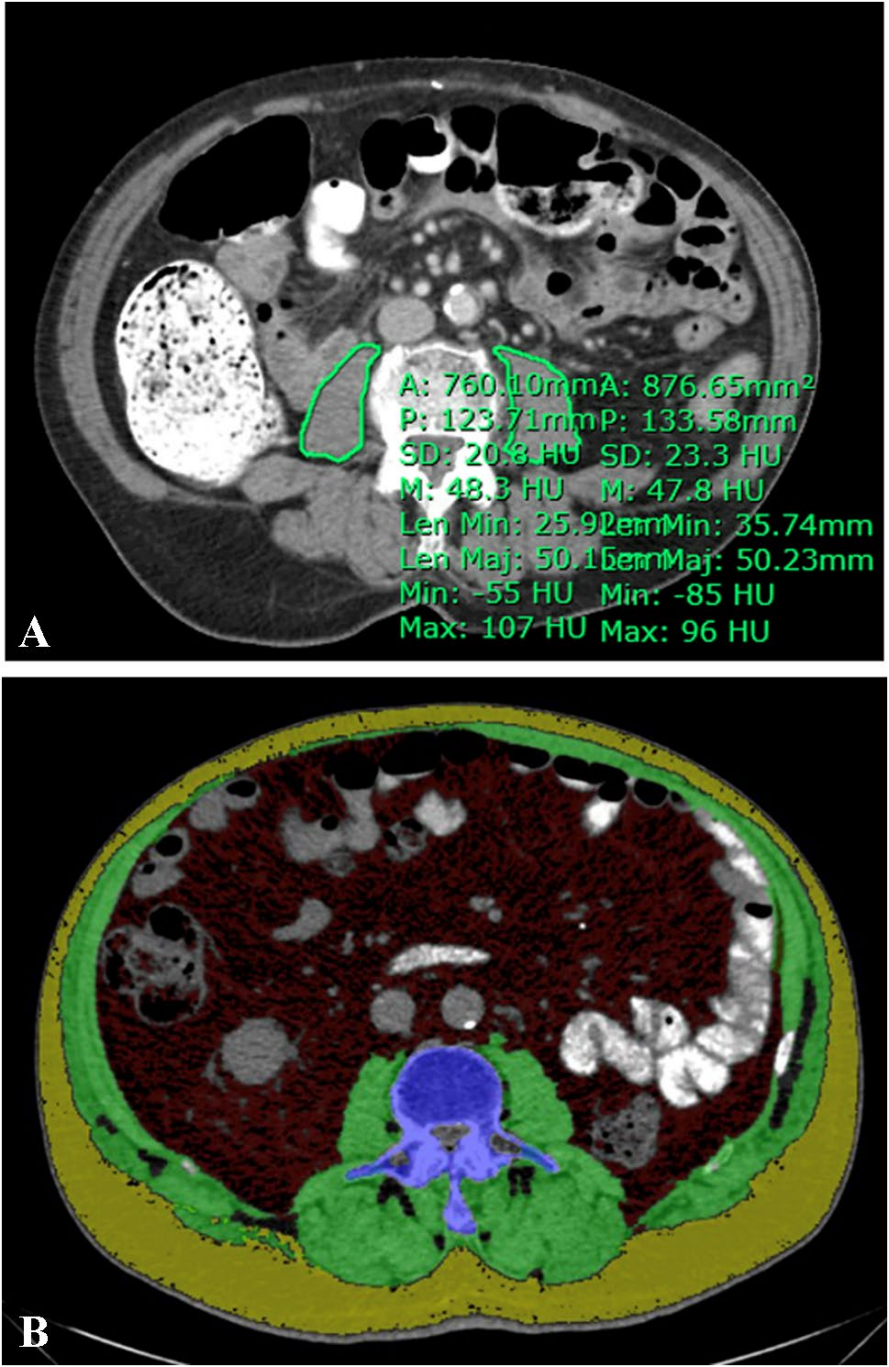
CT image showing the segmentation of psoas muscles (**A**), visceral fat area (**B**-red), and subcutaneous fat are (**B**-yellow)

**Fig. 3:**
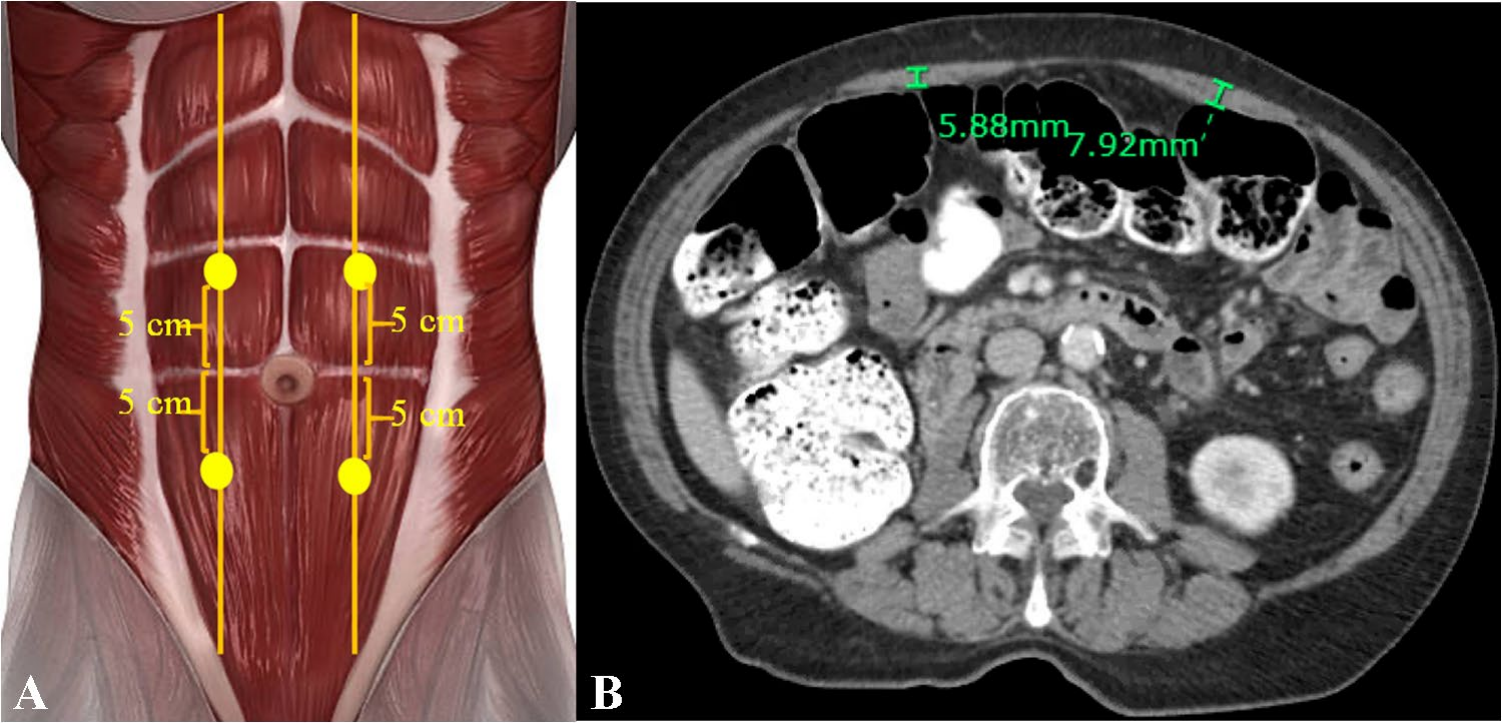
Rectus muscle thickness measurement: schematic location of the 4 points of interest (**A**), axial view of the rectus thickness in right and left upper quadrant points (**B**)

For each patient, pre- and post-operative CT images were analyzed, and the following body composition metrics were extracted. The last CT image (for patients without IH) and the CT at the time of IH development (for patients with IH) were considered as the reference to be compared with the preoperative imaging.

a *Psoas muscle:* cross-sectional psoas muscle mass at the superior margins of L3 and L4 vertebral bodies were measured, as previously described [11]. Using Synapse 3D software (Fujifilm, CT) semiautomated calculation was performed with manual segmentation of the right and left psoas muscles based on the Hounsfield unit (HU) threshold for muscle (- 30 to + 150 HU) (Fig. 2-A). Psoas muscle index (PMI) was calculated as total psoas muscle area divided (cm^2^) by the square of the body height (m^2^) at the L3 and L4 levels. Sarcopenia was defined as PMI below the lowest quartile (Q1), as described before [13]. Two other measures for muscle mass, using Q2 and Q3 cutoffs, were also used to explore the effect of low vs. high PMI in patients.
b *Body fat:* the subcutaneous fat area (SFA) and visceral fat area (VFA) were automatically derived by 2D segmentation at the level of L3 (Fig. 2-B). A CT range of -120 to -40 HUs was considered as a threshold for fat, using semiautomated calculation.
c *Rectus muscle thickness:* this parameter was calculated at four points at the mid-rectus line, 5 cm caudal or cranial to the umbilicus (Fig. 3). Both mean 4-quadrant and 2-quadrant (right or left, according to the surgical side) rectus muscle thickness was used in the final analysis.

### Clinical data collection and outcomes variables

Clinical features, including age, gender, Charlson Comorbidity Index (CCI), American Society of Anesthesiologists (ASA) score, body mass index (BMI), smoking, and underlying chronic obstructive pulmonary disease were captured. Operative and treatment details, including side and type of surgery (partial vs. radical nephrectomy), surgical approach (retroperitoneal vs. transperitoneal), operative time, estimated blood loss, and pathological stage were also recorded. The primary outcome variables were radiological IH and time to radiologically defined IH.

### Statistical analysis

Demographic and clinical features were summarized as counts and percentages for categorical variables and mean ± standard deviation (SD) or median (interquartile range: IQR) for continuous variables. A Cox proportional hazard model was used to examine the risk of various factors, including sarcopenia, SFA, VFA, and rectus muscle thickness for IH. Proportional hazard assumption was assessed by supremum test and Schoenfeld residual plots. For each measurement, we have tested both the linear and non-linear effects. Sex was considered as an effect modifier when tested for the linear effect. Non-linear effect was searched by dichotomizing data using variable specific quartile values. For each measurement, we have generated three dichotomized variables cut by 25^th^ (Q1), median (Q2), and 75^th^ (Q3) percentile, and tested by three separate models. Dichotomizing data by change < 25^th^ vs. ≥ 25^th^ percentile allowed us to detect any “U” shape association between each body composition metric and IH. Multivariable model was applied to adjust the outcomes by potential confounders, including age, sex, and tumor stage. Benjamini-Hochberg (BH) Procedure [21] was used to control multiple comparison error.

Statistical software package SAS, Version 9.4 (SAS Institute Inc., Cary, NC, USA) was applied to all the analyses in this study. All *p* values reported were 2-sided and *p* < 0.05 was considered statistically significant.

## Results

A total of 236 patients were included in this study. Median (IQR) age was 64 (54–70) years, including 169 (72%) and 67 (28%) males and females, respectively. The baseline clinical and surgical features of the patients are shown in Table 1.

**Table 1:** Demographic and clinical data of the patients

Variable	All patients (*n* = 236)	IH (*n* = 62)	No IH (*n* = 174)
Age, median (IQR), year	64 (54–70)	62 (52–71)	64 (55–70)
Sex, n (%)			
Male	169 (72)	39 (63)	130 (75)
Female	67 (28)	23 (37)	44 (25)
ASA, n (%)			
1–2	100 (42)	25 (40)	75 (43)
≥ 3	136 (58)	37 (60)	99 (57)
CCI, n (%)			
0	91 (52)	35 (56)	126 (53)
≥ 1	83 (48)	27 (44)	110 (47)
BMI, median (IQR), Kg/m^2^	28 (24–32)	28 (26–34)	28 (24–32)
Hx of COPD, n (%)	23 (10)	7 (11)	16 (9)
Hx of smoking, n (%)	79 (33)	19 (31)	60 (34)
Type of procedure, n (%)			
Partial nephrectomy	74 (31)	22 (35)	52 (30)
Radical Nephrectomy	162 (69)	40 (65)	122 (70)
Surgical approach, n (%)			
Transperitoneal	219 (93)	58 (94)	161 (93)
Retroperitoneal	17 (7)	4 (6)	13 (7)
Side, n (%)			
Right	128 (54)	33 (53)	95 (55)
Left	108 (46)	29 (47)	79 (45)
Operative time, median (IQR), min	242 (200–303)	262 (209–321)	242 (198–299)
EBL, median (IQR), mL	150 (100–250)	200 (100–300)	150 (100–250)
Pathological stage, n (%)			
≤ T2	193 (82)	50 (81)	143 (82)
> T3	43 (18)	12 (19)	31 (18)

*ASA*: American Society of Anesthesiologists; *CCI*: Charlson comorbidity index; *BMI*: body mass index; *Hx*: History; *COPD*: chronic obstructive pulmonary disease; *EBL*: estimated blood loss

In a median (IQR) follow-up of 23 (14–38) months, 62 (26%) patients developed radiologic IH. Median (IQR) time to radiologic IH was 12.1 (5.7–16.7) months. The location of IH was medial, anterolateral, and posterior in 37 (60%), 21 (34%), and 3 (6%), respectively. IH was graded as early-onset (*n* = 29, 47%), late-onset (*n* = 26, 42%), and bowel-containing (*n* = 7, 11%). The median (IQR) hernia size was 7.8 (4.5–13.5) millimeters.

The distribution of musculoskeletal quantitative CT metrics is shown in Table 2. The non-linear and linear associations between muscle/fat metrics and IH development are shown in Tables 3 and 4, respectively. The cutoff values of PMI used for assessment of sarcopenia at L3 and L4 levels were 4.20 and 6.14 cm^2^/m^2^, respectively. Using these data driven cutoffs on Cox proportional hazard model, we did not find a statistically significant non-linear association between sarcopenia and radiologic IH. However, the preoperative PMI below 75% percentile (Q3) at L4 has statistically significant risk of radiologic IH with a hazard ratio (HR) of 2.27 (95% CI 1.11, 4.54; *p* = 0.02). At L3, the baseline PMI below median (Q2) has borderline statistically significant risk of radiologic IH with a HR of 1.63 (95% CI 0.98, 2.7; *p* = 0.06). After adjusting for age, sex, and tumor stage, only PMI < Q3 at the level of L4 showed borderline statistically significant association with IH (HR 2.04 [95% CI 0.96, 4.35]; *p* = 0.06).

**Table 2:** Distribution of radiologic musculoskeletal metrics

Variable	Cutoffs		
	Quartile 1	Quartile 2	Quartile 3
Preop PMI at L3, cm^2^/m^2^	4.20	6.04	7.31
Preop PMI at L4, cm^2^/m^2^	6.14	7.51	9.05
Subcutaneous fat area change, cm^2^	-13.52	5.50	22.27
Visceral fat area change, cm^2^	-30.47	3.12	26.83
Mean 4-Q rectus muscle thickness change, mm	-0.7	-0.07	0.35
Mean 2-Q rectus muscle thickness change, mm	-0.75	-0.1	0.4

*PMI*: Psoas Muscle Index; *Q*: quadrant

**Table 3:** The non-linear association of change in musculoskeletal metrics from pre to post scan with hernia development^*^

Measurements	Reference	Univariate		Multivariable^#^	
		HR (95% CI)	*p* value	HR (95% CI)	*p* value
Preop Psoas Muscle Index (PMI)					
L3 Level	< Q1 vs ≥ Q1	1.25 (0.71, 2.18)	0.44	0.92 (0.48, 1.8)	0.82
L3 Level	< Q2 vs ≥ Q2	1.63 (0.98, 2.7)	0.06	1.4 (0.78, 2.52)	0.26
L3 Level	< Q3 vs ≥ Q3	1.10 (0.61, 1.96)	0.76	0.91 (0.48, 1.72)	0.77
L4 Level	< Q1 vs ≥ Q1	1.24 (0.72, 2.15)	0.44	0.84 (0.42, 1.68)	0.63
L4 Level	< Q2 vs ≥ Q2	1.62 (0.98, 2.7)	0.06	1.46 (0.79, 2.67)	0.22
L4 Level	< Q3 vs ≥ Q3	2.27 (1.11, 4.54)	0.02	2.04 (0.96, 4.35)	0.06
Mean Rectus Muscle Thickness Change from Preop					
2-Quadrant	< Q1 vs ≥ Q1	0.99 (0.54, 1.79)	0.97	1.03 (0.57, 1.88)	0.92
2-Quadrant	< Q2 vs ≥ Q2	0.67 (0.41, 1.12)	0.13	0.65 (0.39, 1.08)	0.1
2-Quadrant	≥ Q3 vs < Q3	1.71 (1, 2.91)	0.05	1.98 (1.14, 3.44)	0.02
4-Quadrant	< Q1 vs ≥ Q1	0.86 (0.47, 1.59)	0.63	0.85 (0.46, 1.57)	0.61
4-Quadrant	< Q2 vs ≥ Q2	0.68 (0.41, 1.14)	0.14	0.62 (0.37, 1.05)	0.07
4-Quadrant	≥ Q3 vs < Q3	2.02 (1.2, 3.41)	< 0.001	2.27 (1.33, 3.87)	< 0.001^†^
Fat Area Change from Preop					
Subcutaneous	< Q1 vs ≥ Q1	2.17 (1.3, 3.64)	< 0.001	2.01 (1.18, 3.44)	0.01
Subcutaneous	< Q2 vs ≥ Q2	0.72 (0.44, 1.19)	0.02	0.72 (0.43, 1.21)	0.21
Subcutaneous	≥ Q3 vs < Q3	2.52 (1.51, 4.23)	< 0.001	2.43 (1.42, 4.14)	< 0.001^†^
Visceral	< Q1 vs ≥ Q1	2.43 (1.44, 4.1)	< 0.001	2.8 (1.62, 4.87)	< 0.001^†^
Visceral	< Q2 vs ≥ Q2	1.12 (0.68, 1.84)	0.67	1.18 (0.71, 1.98)	0.52
Visceral	≥ Q3 vs < Q3	1.83 (1.08, 3.08)	0.02	1.79 (1.06, 3.04)	0.03

^#^ Adjusted by age, sex, and tumor stage, *Q*: quartile

^†^ Remained as statistically significant after adjustments

^*^ For each measurement, three dichotomized variables cut by 25^th^ (Q1), median (Q2), and 75^th^ (Q3) percentile were generated and tested by three separate models

**Table 4:** The linear association of change in musculoskeletal metrics from pre to post scan with hernia development

Measurements	HR (95% CI), *p value*			Interaction *p* value
	Overall	Male	Female	
Preop PMI at L3, cm^2^/m^2^	0.86 (0.63, 1.16), 0.31	0.78 (0.55, 1.11), 0.17	1.09 (0.64, 1.84), 0.75	0.29
Preop PMI at L4, cm^2^/m^2^	0.78 (0.57, 1.08), 0.14	0.71 (0.49, 1.03), 0.07	1.04 (0.57, 1.88), 0.9	0.27
Subcutaneous fat area change, cm^2^	0.9 (0.78, 1.03), 0.13	0.9 (0.78, 1.04), 0.17	0.71 (0.27, 1.88), 0.49	0.63
Visceral fat area change, cm^2^	0.86 (0.76, 0.97), 0.01	0.86 (0.76, 0.98), 0.02	0.56 (0.19, 1.71), 0.31	0.46
Mean 4-Q rectus muscle thickness change, mm	1.29 (1.04, 1.58), 0.02	1.18 (0.91, 1.54), 0.21	2.95 (1.53, 5.66), < 0.01	0.01
Mean 2-Q rectus muscle thickness change, mm	1.28 (0.8, 2.04), 0.3	0.91 (0.56, 1.47), 0.69	1.73 (1.14, 2.6), < 0.01	0.05

*PMI*: Psoas muscle index; *Q*: Quadrant

In terms of SFA change from pre-op, both lower and higher values (i.e., < Q1 vs. ≥ Q1, and ≥ Q3 vs. < Q3) were associated with IH development (HR 2.17 [95% CI 1.33, 3.64] and 2.52 [1.51, 4.23], respectively). The association remained significant after adjusting for potential confounders, including age, sex, and tumor stage (HR 2 [1.18, 3.44] and 2.4 [1.42, 4.14], respectively). Similar trend was found for VFA changes from pre-op with a HR of 2.4 [1.42, 4.1] for < Q1 vs. others and 1.8 [1.08, 3.08] for ≥ Q3 vs. others. Again, the association remained statistically significant on multivariable analysis (HR 2.8 [1.62, 4.87] and 1.8 [1.06, 3.04], respectively).

Regarding rectus muscle, increase in the mean 4-quadrant rectus muscle thickness from pre-op was associated with a risk of IH development (HR: 2.3 [1.33, 3.87] for ≥ Q3 [0.35 mm], *p* < 0.001). The same trend was observed for mean change in 2-quadrant rectus muscle thickness (HR 2 [1.2, 3.41] for ≥ Q3 [0.4 mm], *p* = 0.02).

## Discussion

The results of our study showed the important role of both subcutaneous and visceral fat change on IH development in patients who undergo robotic nephrectomy. However, preoperative sarcopenia was not associated with increased risk of hernia development.

Sarcopenia is an important part of body composition analysis that can potentially generate objective anthropometric information to help with prognostication and treatment decisions in urological surgeries [21]. It can be calculated using cross-sectional images, including CT and magnetic resonance imaging that is usually available as part of the preoperative workup. There is no consensus on the best method of evaluating sarcopenia. Studies have used different indices in this regard, including thoracic muscle density/index, skeletal muscle index/density, and unilateral/bilateral psoas muscle area/index. The two latter can be measured at the L3 or L4 levels using different cutoffs [11, 22]. Despite recent advances in software and machine learning models that allow for fully automated deep learning muscle quantitation, these measurements have been commonly performed through semi-automated methods (i.e., segmentation by a trained human analyst) [21, 23]. In this study, we used PMI (as the surrogate for lean core muscle mass) for the evaluation of sarcopenia and calculated this index at both L3 and L4 levels in a semi-automated fashion.

Sarcopenia has emerged as a potential prognostic factor in different types of surgeries. It has been shown to be an independent predictor of poor postoperative outcomes following major abdominal, particularly oncological, surgeries. In recent studies, sarcopenia has been associated with increased rates of infection, length of hospital stay, morbidity, mortality, and readmission, as well as hospital costs [24, 25]. Nevertheless, limited data is available regarding the association between sarcopenia and incisional hernia. In a study of 283 patients who underwent elective midline laparotomy, van Roojien et al. reported no association between sarcopenia and the development of IH. They assessed sarcopenia using skeletal muscle index (SMI) that has been measured at the level of L3 in the preoperative CT scan [13]. In addition, a recent study on patients undergoing appendectomy reported that psoas’ CT attenuation was an independent protective factor for IH, yet PMI and sarcopenia did not show a significant effect on hernia development [26]. Only a few other studies have evaluated the role of sarcopenia in patients undergoing hernia repair. However, most of these studies included heterogenous group of patients, used different types of measurement for sarcopenia, and reported mixed results [12, 14, 17, 18]. In a retrospective study including 58 patients undergoing ventral hernia repair, preoperative sarcopenia was associated with an increased risk for postoperative complications, including higher rate of hernia recurrence [14]. Nevertheless, another study on 135 patients who underwent the same type of surgery showed that sarcopenia was not associated with an increase in postoperative complications, surgical site infections, or hernia recurrence [18]. A recent systematic review evaluating the effect of sarcopenia on ventral hernia/abdominal wall repair identified important factors contributing to the heterogeneity in results. These factors mainly include inconsistency in chosen methodology as well as the outdated definitions of sarcopenia [23]. Unlike the previous studies that evaluated muscle indices at either L3 or L4, we calculated PMI at both levels. Our data showed that in patients undergoing robotic nephrectomy, preoperative sarcopenia is not an independent risk factor of IH. However, using other cutoffs, we found that the preoperative PMI below 75% percentile at the level of L4 increases the risk of radiologic IH by more than twofold (HR 2.27, *p* = 0.02). Moreover, this effect was similar when considering PMI below 50% percentile at the L3 level (HR 1.63), although the *p* value was not significant.

Body fat volume has also been shown to be associated with IH development. In a study of 193 patients undergoing colorectal cancer resection, Aquina et al. showed that visceral obesity but not BMI was a significant risk factor for IH (HR 2.04). They concluded that visceral fat volume is a more appropriate proxy for central obesity compared to BMI [12]. Similar association was found in our study with a 1.8-fold increased risk of IH in patients who had higher visceral fat gain. This might be due to the increased abdominal wall tension and/or intra-abdominal pressure in obese patients. In our study, we also noticed an increased risk of IH in patients who lose visceral fat following surgery (HR 2.17). In these patients, both frailty and loss of abdominal wall support can contribute to the development of IH. We found similar trends for subcutaneous fat area, indicating increased risk of IH in both subcutaneous fat gain and loss conditions.

Rectus muscle thickness can potentially affect the occurrence of hernia. A recent study on patients who underwent end colostomy showed that atrophy of the left lower medial section of the abdominal rectus muscle is associated with an increased risk of parastomal hernia [27]. In our study, increasing rectus muscle thickness was associated with an increased risk of IH. This was statistically significant for mean 4-quadrant and 2-quadrant rectus muscle thickness changes from pre-op. This is contrary to our hypothesis on the protective role of rectus muscle in hernia development. Our findings can be explained by the scar tissue formation and rectus muscle pseudohypertrophy that may develop after robotic nephrectomy due to port placements. Moreover, despite this significant statistical association, the amount of change (i.e., submillimeter) is exceedingly small from the clinical standpoint.

The exploration of risk factors contributing to the development of IH is important as they can be considered in preoperative counseling in patients undergoing surgery, especially cancer patients who suffer from various comorbidities. In line with the previous studies [28, 29], we reported a 26% incidence of radiological IH in our cohort. This high rate of IH can negatively affect the quality of life of patients and impose a significant financial burden as it requires several clinic visits and possible surgical repair. Obesity and frailty, as modifiable risk factors of IH, can potentially be mitigated with proper patient optimization pre- and postoperatively. This goal can be achieved through combination of exercise and diet modification that might improve the body fat distribution as well as functional status of the patients [30].

An important limitation of this study is its retrospective nature that may affect the accuracy of select subjective variables. In addition. since IH is a not an acute condition, the true onset of IH could be earlier than time of radiological appearance during routine follow-up. However, our study is robust as it included a large patient cohort, compared to the literature, who underwent standardized surgery by experienced urologists. In addition, all follow-up images were re-evaluated by an experienced radiology team.

## Conclusion

Both excessive body fat gain and loss could be associated with development of radiologic incisional hernia in patients undergoing robotic nephrectomy. Nevertheless, no association was found between sarcopenia and risk of hernia development. Prospective studies with larger sample size are needed to confirm the results of our study.
